# Genetic and antigenic characterisation of influenza A(H3N2) viruses isolated in Yokohama during the 2016/17 and 2017/18 influenza seasons

**DOI:** 10.2807/1560-7917.ES.2019.24.6.1800467

**Published:** 2019-02-07

**Authors:** Chiharu Kawakami, Seiya Yamayoshi, Miki Akimoto, Kazuya Nakamura, Hideka Miura, Seiichiro Fujisaki, David J. Pattinson, Kohei Shimizu, Hiroki Ozawa, Tomoko Momoki, Miwako Saikusa, Atsuhiro Yasuhara, Shuzo Usuku, Ichiro Okubo, Takahiro Toyozawa, Shigeo Sugita, Derek J. Smith, Shinji Watanabe, Yoshihiro Kawaoka

**Affiliations:** 1 Yokohama City Institute of Public Health, Yokohama, Japan; 2 Division of Virology, Department of Microbiology and Immunology, Institute of Medical Science, University of Tokyo, Tokyo, Japan; 3 Influenza Virus Research Center, National Institute of Infectious Diseases, Tokyo, Japan; 4 Center for Pathogen Evolution, University of Cambridge, Cambridge, UK; 5 Yokohama City Public Health Center, Yokohama, Japan; 6 Equine Research Institute, Japan Racing Association, Tochigi, Japan; 7 Department of Pathobiological Sciences, School of Veterinary Medicine, University of Wisconsin-Madison, USA; 8 Department of Special Pathogens, International Research Center for Infectious Diseases, Institute of Medical Science, University of Tokyo, Tokyo, Japan

**Keywords:** H3N2, HA, haemagglutinin, antigenicity, glycosylation, Japan, viral infections, influenza, influenza virus, surveillance, epidemiology

## Abstract

Background: Influenza A(H3N2) virus rapidly evolves to evade human immune responses, resulting in changes in the antigenicity of haemagglutinin (HA). Therefore, continuous genetic and antigenic analyses of A(H3N2) virus are necessary to detect antigenic mutants as quickly as possible.

Aim: We attempted to phylogenetically and antigenically capture the epidemic trend of A(H3N2) virus infection in Yokohama, Japan during the 2016/17 and 2017/18 influenza seasons.

Methods: We determined the HA sequences of A(H3N2) viruses detected in Yokohama, Japan during the 2016/17 and 2017/18 influenza seasons to identify amino acid substitutions and the loss or gain of potential N-glycosylation sites in HA, both of which potentially affect the antigenicity of HA. We also examined the antigenicity of isolates using ferret antisera obtained from experimentally infected ferrets.

Results: Influenza A(H3N2) viruses belonging to six clades (clades 3C.2A1, 3C.2A1a, 3C.2A1b, 3C.2A2, 3C.2A3 and 3C.2A4) were detected during the 2016/17 influenza season, whereas viruses belonging to two clades (clades 3C.2A1b and 3C.2A2) dominated during the 2017/18 influenza season. The isolates in clades 3C.2A1a and 3C.2A3 lost one N-linked glycosylation site in HA relative to other clades. Antigenic analysis revealed antigenic differences among clades, especially clade 3C.2A2 and 3C.2A4 viruses, which showed distinct antigenic differences from each other and from other clades in the antigenic map.

Conclusion: Multiple clades, some of which differed antigenically from others, co-circulated in Yokohama, Japan during the 2016/17 and 2017/18 influenza seasons.

## Introduction

Influenza A(H3N2) virus has continued to infect humans since its emergence as a pandemic virus in 1968, resulting in considerable economic burden, hospitalisations and deaths [1]. After half a century of circulating in humans, A(H3N2) virus has accumulated numerous amino acid substitutions in its haemagglutinin (HA) to escape from human antibodies against this protein. Mouse monoclonal antibodies identified five major antigenic sites, A through E, on HA [2,3] and amino acid substitutions at these major antigenic sites are associated with antigenic drift. Based on the antigenicity of HA, A(H3N2) viruses form antigenic clusters [4]. Seven positions i.e. 145 at antigenic site A and 155, 156, 158, 159, 189 and 193 at antigenic site B, are mainly responsible for antigenic cluster transitions [5]. In addition, modification of HA with N-linked glycans also affects the antigenicity of HA via steric hindrance at these antigenic sites [6,7].

Seven A(H3N2) clades (designated clades 1 to 7) and many subclades have evolved since 2009. Between 2011 and 2012, clade 3 viruses dominated and formed subgroups, clades 3A, 3B and 3C [8]; clade 3C viruses evolved further and subdivided into clades 3C.1, 3C.2 and 3C.3 [9] In 2014, three new genetic subgroups emerged 3C.2A, 3C.3A and 3C.3B [10].

During the 2014/15 influenza season, the majority of reported influenza infections in Japan were caused by A(H3N2) viruses of clade 3C.2A [11], whereas in the 2015/16 influenza season only a few infections caused by A(H3N2) virus were reported [12]. Therefore, the 2016/17 and 2017/18 influenza vaccines contained antigens from a virus of clade 3C.2A [13]. Here, we analysed the HA sequences of A(H3N2) viruses detected in Yokohama, Japan during the 2016/17 and 2017/18 influenza seasons to capture the epidemic trend of A(H3N2) virus infection.

## Methods

### Study samples

Clinical specimens were collected in sentinel clinics and hospitals as part of the national epidemiological surveillance of infectious diseases in Japan during the 2016/17 and 2017/18 influenza seasons. These specimens were tested by reverse transcription (RT)-quantitative PCR (RT-qPCR) targeting H3-HA gene [14] and virus isolation was achieved by using AX4 cells.

### Cells and culture

AX4 cells and (Madin-Darby canine kidney (MDCK)-β-galactoside α2,6-sialyltransferase I (SIAT1) cells, which express higher amounts of six-linked sialic acids on their cell surface via exogenous expression of human SIAT1 (or ST6Gal I) [15,16] were maintained in Eagle’s minimal essential medium (MEM) containing 10% fetal calf serum (FCS) and Dulbecco’s modified eagle medium (DMEM) containing 5% fetal calf serum and 1 mg/mL G418 sulphate (ThermoFisher Scientific, Tokyo, Japan), respectively. Both cell lines were incubated at 37 °C under 5% CO_2_ and were passaged by the standard procedure.

### Viruses

Influenza A(H3N2) viruses A/Gunma/140/2017, A/Kagoshima/74146/2017, A/Osaka/163/2017, A/Shimane/112/2017, A/Aichi/343/2017 and A/Okinawa/64/2017 were obtained from Gunma Prefectural Institute of Public Health and Environmental Sciences, Kagoshima Prefectural Institute for Environmental Research and Public Health, Osaka Institute of Public Health, Shimane Prefectural Institute of Public Health and Environment Science, Aichi Prefectural Institute of Public Health, and Okinawa Prefectural Institute of Health and Environment, respectively.

#### Sequence analysis

Viral RNA was extracted from the isolated viruses by using an RNeasy Mini Kit (QIAGEN, Tokyo, Japan). The viral RNA was subjected to one step RT-PCR to amplify the HA gene by PCR using the AccessQuick RT-PCR system (Promega, Madison, Wisconsin, United States of America (USA)) as follows: after 45 minutes of cDNA synthesis at 48 °C and 2 minutes of denaturation at 94 °C, samples were subjected to 40 cycles of amplification, consisting of 1 minute at 94 °C, 90 seconds at 55 °C and 2 minutes at 68 °C, with a final additional extension step at 68 °C for 10 minutes. The PCR products were purified with a QIAquick PCR Purification Kit (QIAGEN, Tokyo, Japan) and then sequenced using a BigDye Terminator v1.1 cycle sequencing kit (Applied Biosystems, Foster City, California (CA), USA) on an ABI 3500 sequencer (Applied Biosystems, Foster City, CA, USA).

### Phylogenetic analysis

The phylogenetic tree was constructed by using the neighbour-joining method with Kimura distances and the bootstrap procedure (n = 1,000) using ClustalW 2.1 on the DDBJ (DNA Data Bank of Japan) website (http://clustalw.ddbj.nig.ac.jp/) and was visualised by using the MEGA 7.0.26 software. A/Perth/16/2009 is defined as an outgroup. HA sequences of reference isolates were obtained from the Global Initiative on Sharing Avian Influenza Data (GISAID) EpiFlu database (https://www.gisaid.org/). The sequencing dataset used in this study is available upon request.

### Three-dimensional mapping

Amino acid positions were plotted on an HA trimer of A/Victoria/361/2011 (PDB ID code 4O5N) by using the Python-enhanced molecular (PyMOL) molecular graphics system version 1.3. The N-glycans on the HA trimer were added to the N-linked glycosylation sites by using the GlyProt web server (http://www.glycosciences.de/modeling/glyprot/php/main.php) and visualised by using the PyMOL software.

### Ferret antisera

The ferret antisera were produced at an animal production and supply company (Japan SLC, Inc. [17]), with the approval of Japan’s Institutional Animal Care and Use Committee of the National Institute of Infectious Diseases. Adult male ferrets that were serologically negative for influenza viruses were infected with the indicated A(H3N2) virus via the respiratory tract as a viral vapour generated by a nebuliser. At 2 weeks after infection, whole blood collected from the ferrets under anaesthesia was coagulated. The serum supernatants after centrifugation were collected as the antiserum. The serum samples were treated with receptor destroying enzyme (RDE) II (SEIKEN, Niigata, Japan), according to the manufacturer’s instructions before use in the virus micro-neutralisation assay.

### Antigenic analysis

An in vitro virus micro-neutralisation assay, based on the concept of plaque reduction, was conducted. Briefly, after 50 μl of twofold serial dilutions of the ferret antisera in DMEM containing 200 nM oseltamivir was added to a monolayer of MDCK-SIAT1 cells in a 96-well plate, 1,000 focus-forming units of the indicated virus (50 µl) was added to each well. After a 1 hour incubation, 1.6% Avicel RC-581 (FMC BioPolymer) in DMEM was overlaid. Oseltamivir was used to prevent receptor binding via NA, and trypsin was not added to avoid virus multiplication. At 18‒20 hours after infection, the cells were stained with mouse anti-nucleoprotein (NP) monoclonal antibodies (MAB8257 and MAB8258; Millipore, Consett, US), followed by an anti-mouse IgG antibody conjugated with horseradish peroxidase. NP-positive cells were visualised with KPL TrueBlue substrate (Kirkegaard and Perry Laboratories) to count foci. The reciprocal number of the minimum dilution of sera needed to achieve 50% focus reduction was used as the neutralisation titre.

### Antigenic cartography

The neutralisation data were analysed with antigenic cartography, which is a method to visualise and increase the resolution of neutralisation results, as detailed previously [4,18]. Diagnostic plots showed that three dimensions and a column basis titre of 1,280 were suitable for representing these neutralisation data.

### Ethical statement

All experiments with ferrets were performed in accordance with the institutional Regulations for Animal Care and Use and were approved by the Japan’s Institutional Animal Care and Use Committee of the National Institute of Infectious Diseases.

## Results

### A(H3N2) viruses circulating in Yokohama, Japan

**Figure 1:**
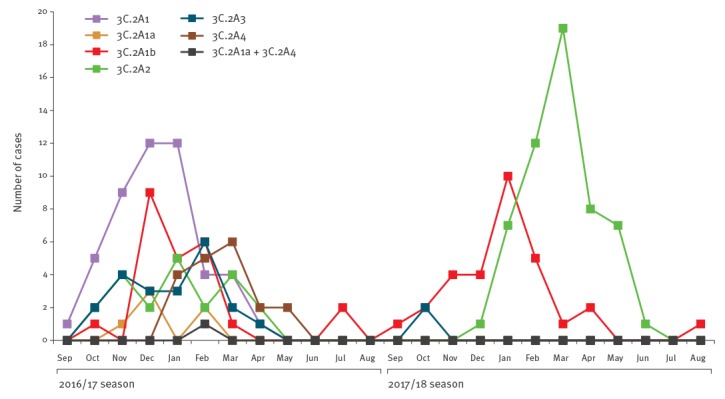
Number of influenza A(H3N2) influenza cases, Yokohama, 2016/17 and 2017/18 influenza seasons

**Figure 2:**
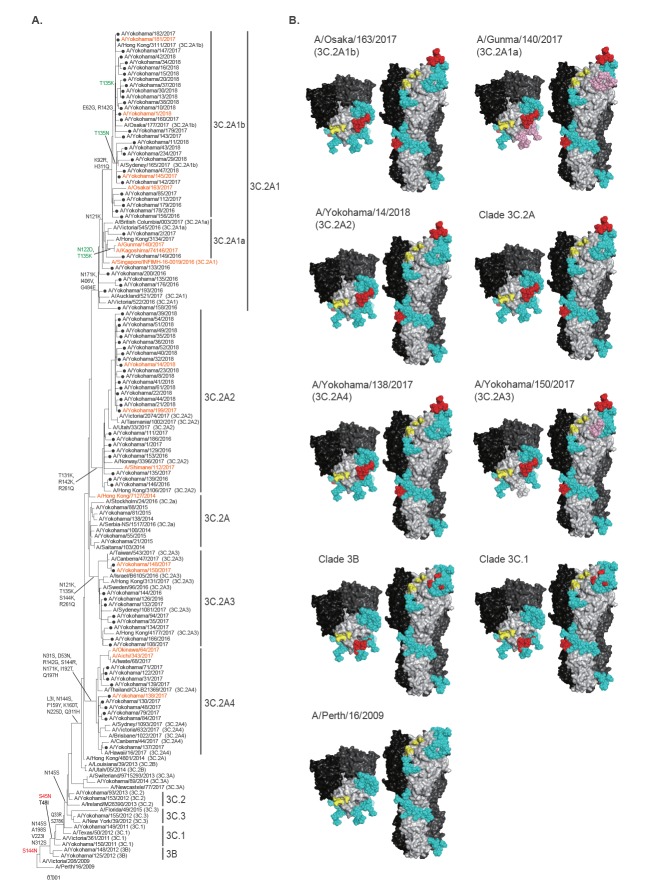
(A) Phylogenic tree of the haemagglutinin (HA) sequences of isolates^a^ and (B) Alteration of N-linked glycosylation on H3-HA^b^, Yokohama, 2016/17 and 2017/18 influenza seasons

HA: haemaglutinin; NA: neuraminidase; PyMOL: Python-enhanced molecular.

^a^The evolutionary history was inferred using the neighbour-joining method with Kimura distances. The viruses isolated in this study are indicated by black circles. The clade of the reference isolates, which are available in the GISAID EpiFlu database, is indicated in parentheses. Amino acid substitutions delineating major branches are shown. Amino acid changes involved in loss or gain of N-glycosylation are shown in green or red, respectively. The isolates used for antigenic analysis are shown in orange. A/Perth/16/2009 is defined as an outgroup.

^b^N-linked glycans present on all of the HAs on the HA trimer of A/Victoria/361/2011 (PDB ID code 4O5N) were added to the N-linked glycosylation sites in the crystal structure by using the GlyProt web server and were visualised by using the molecular graphics system PyMOL. Consensus sequences of clades 3B, 3C.1 and 3C.2A, and representative isolates of each clade, were used. Newly emerged N-linked glycans at N45, N144 and N158 are shown in red and the lost glycans at N122 and N133 are shown in light pink. Other N-linked glycans are shown in cyan. The receptor-binding site is shown in yellow.

During the 2016/17 and 2017/18 influenza seasons, we detected 227 A(H3N2) viruses from clinical samples collected in Yokohama; 225 viruses were isolated using AX4 cells and two were positive by RT-qPCR (Figure 1). The HA sequences were determined by Sanger sequencing and HA genetic clades were determined based on reference isolates available through EpiFlu To draw the phylogenetic tree using the neighbour-joining method with Kimura distances, we selected several isolates as representative of each clade. We found that A(H3N2) viruses isolated during the 2016/17 and 2017/18 influenza seasons in Yokohama could be classified into six clades: 3C.2A1, 3C.2A1a, 3C.2A1b, 3C.2A2, 3C.2A3, and 3C.2A4 (Figure 1 and Figure 2A), based on the World Health Organization (WHO) designations. We considered one case to be a co-infection with A(H3N2) viruses of two different clades because we detected HA sequences of clades 3C.2A1a and 3C.2A4. Isolates of all six clades were detected during the 2016/17 influenza season, whereas several isolates of clades 3C.2A1b, 3C.2A2, and 3C.2A3 and no isolates of clades 3C.2A1, 3C.2A1a and 3C.2A4 were detected during the 2017/18 influenza season (Figure 1). These results demonstrate that several different clades of A(H3N2) viruses simultaneously caused influenza in Yokohama and the diversity of the circulating clades differed between the 2016/17 and the 2017/18 influenza seasons; the vaccine strain for A(H3N2) virus component was identical between two seasons, A/Hong Kong/4801/2014 [13].

The number of cases summarised based on the haemagglutinin (HA) clades, assigned according to World Health Organization reports.

### Changes in the N-linked glycosylation sites on H3-HA

We next focused on the putative N-linked glycosylation sites on the H3-HA molecule because gradual accumulation of putative N-linked glycosylation sites has been linked to escape from human antibody pressure [19]. On a single HA molecule of A/Perth/16/2009, we found 11 putative N-linked glycosylation sites (Figure 2B). Clade 3B or 3C.1 viruses acquired one or two putative N-linked glycosylation sites at positions 144‒146 (NSS) or 45‒47 (NSS) and 144‒146 (NSS) due to the S45N or S144N substitution. Among the clade 3C.2A viruses, the N144S substitution and the K160T substitution abolished and created the putative N-linked glycosylation site at positions 144‒146 and 158‒160 (NYT), respectively. The N-linked glycosylation sites of clade 3C.2A viruses were conserved among isolates of clades 3C.2A1b, 3C.2A2 and 3C.2A4, whereas some viruses in clade 3C.2A1b possessed the T135K or T135N substitution causing the loss of the glycosylation site at positions 133‒135 (NGK or NGN) and the gain of the glycosylation site at positions 135‒137 (NSS). Several isolates of clade 3C.2A1a lost two putative N-linked glycosylation sites at positions 122‒124 (NES) and 133–135 (NGT) due to the N122D and T135K substitutions. Isolates of clade 3C.2A3 lost a putative N-linked glycosylation site at position 135 (NGT) as a result of the T135K substitution. Thus, viruses with HA that possessed different N-linked glycosylation sites, which possibly affected antigenicity, co-circulated.

### Antigenicity analysis

**Figure 3:**
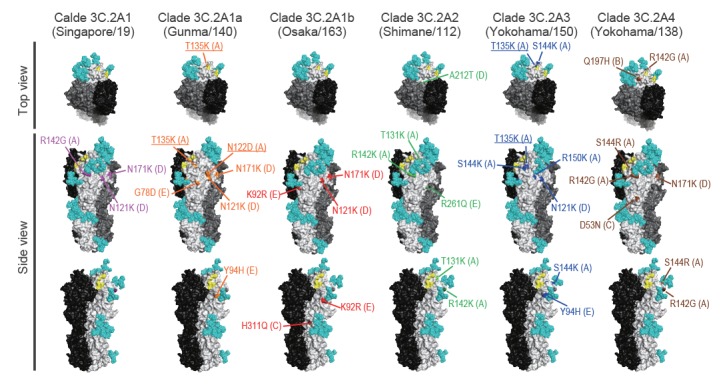
Amino acid substitution sites mapped onto the H3-HA molecule, Yokohama, 2016/17 and 2017/18 influenza seasons

Since the phylogenetic tree revealed that A(H3N2) viruses that were distinct in terms of their HA sequence and number of HA glycosylation sites co-circulated in Yokohama during the 2016/17 and 2017/18 influenza seasons, we examined the antigenicity of viruses from different clades using sera obtained from infected ferrets. We prepared ferret antisera by infecting ferrets with each of five influenza A(H3N2) virus isolates: A/Hong Kong/7127/2014 (clade 3C.2A), which is antigenically similar to A/Hong Kong/4801/2014 (clade 3C.2A), the vaccine seed virus for the northern hemisphere 2016/17 and 2017/18 influenza seasons; A/Singapore/INFIMH-16–0019/2016 (clade 3C.2A1), the vaccine seed virus for the 2018 southern and 2018/19 northern hemisphere influenza season; A/Osaka/163/2017 as a representative of clade 3C.2A1b; A/Shimane/112/2017, as a representative of clade 3C.2A2; and A/Yokohama/138/2017 as a representative of clade 3C.2A4 (Figure 2A). For the antigenicity analysis, we selected 16 isolates (Table 1). We compared the HA sequence of the selected isolates with that of A/Hong Kong/7127/2014 (Hong Kong/7127, clade 3C.2A) at antigenic sites A through E [2,3] and mapped them onto the H3-HA molecule (Figure 3 and Table 2). All six isolates possessed one or two substitutions at antigenic site D. An amino acid substitution at antigenic site A or E was found in five or four isolates, whereas that at antigenic site B or C was found in one or two isolates. A/Kagoshima/74146/2017, A/Yokohama/199/2017, A/Yokohama/14/2018, A/Yokohama/148/2017, A/Aichi/343/2017 and A/Okinawa/64/2017 possessed identical HA antigenic sites to those of other viruses of the same clade. A/Yokohama/181/2017 and A/Yokohama/1/2018 possessed three additional substitutions (E62G, T135K, and R142G) at antigenic sites E, A and A, respectively. A/Yokohama/145/2017 possessed one additional substitution (T135N) at antigenic site A. Both the T135K and T135N mutations abolished the potential N-glycosylation site at positions 133–135 and T135N mutation generated the glycosylation site at positions 135‒137. All of the tested viruses were isolated and passaged using MDCK cells or derivative AX4 or MDCK-SIAT1 cells.

**Table 1:** Antigenic analysis by neutralisation assay using infected ferret sera, Yokohama, 2016/17 and 2017/18 influenza seasons

Strain	Clade	Neutralisation titres of ferret sera against				
		Hong Kong/7127	Singapore/19	Osaka/163	Shimane/112	Yokohama/138
A/Hong Kong/7127/2014	3C.2A	**320^a^**	80	160	80	80
A/Singapore/INFIMH-16–0019/2016	3C.2A1	320	**160^a^**	160	160	160
A/Gunma/140/2017	3C.2A1a	160	320	80	160	80
A/Kagoshima/74146/2017	3C.2A1a	160	160	80	80	80
A/Osaka/163/2017	3C.2A1b	80	40	**320^a^**	40	40
A/Yokohama/181/2017	3C.2A1b	80	160	80	80	80
A/Yokohama/1/2018	3C.2A1b	160	160	80	80	80
A/Yokohama/145/2017	3C.2A1b	80	160	80	80	80
A/Shimane/112/2017	3C.2A2	320	160	160	**1,280^a^**	160
A/Yokohama/199/2017	3C.2A2	640	320	160	2,560	320
A/Yokohama/14/2018	3C.2A2	640	160	160	2,560	160
A/Yokohama/150/2017	3C.2A3	160	320	160	160	80
A/Yokohama/148/2017	3C.2A3	320	160	160	320	80
A/Yokohama/138/2017	3C.2A4	320	320	160	80	**640^a^**
A/Aichi/343/2017	3C.2A4	320	80	80	40	640
A/Okinawa/64/2017	3C.2A4	320	160	80	40	640

^a^Homologous combinations are shown in bold.

HA: haemaglutinin.

Amino acid substitutions at the antigenic sites of H3-HA derived from A/Singapore/INFIMH-16–0019/2016, A/Gunma/140/2017, A/Osaka/163/2017, A/Shimane/112/2017, A/Yokohama/150/2017 or A/Yokohama/138/2017, compared with A/Hong Kong/7127/2014 (Clade 3C.2A), were mapped onto the HA trimer and are indicated by purple, orange, red, green, blue and brown, respectively, with the antigenic sites in parentheses. High-mannose type N-linked glycans (shown in cyan) on the HA trimer of A/Victoria/361/2011 (PDB ID code 4O5N) have been added. Amino acid substitutions involved in the loss of an N-glycosylation site are underlined. The receptor-binding site is shown in yellow.

**Table 2:** Amino acid differences in antigenic sites A‒E, Yokohama, 2016/17 and 2017/18 influenza seasons

Strain	Clade	Amino acid difference compared with A/Hong Kong/7127/2014 in the antigenic site				
		A	B	C	D	E
A/Singapore/INFIMH-16–0019/2016	3C.2A1	R142G	Null	Null	N121K, N171K	Null
A/Gunma/140/2017	3C.2A1a	N122D, T135K	Null	Null	N121K, N171K	G78D, Y94H
A/Osaka/163/2017	3C.2A1b	Null**^a^**	Null	H311Q	N121K, N171K	K92R
A/Yokohama/1/2018	3C.2A1b	T135K, R142G	Null	H311Q	N121K, N171K	E62G, K92R
A/Yokohama/145/2017	3C.2A1b	T135N	Null	H311Q	N121K, N171K	K92R
A/Shimane/112/2017	3C.2A2	T131K, R142K	Null	Null	A212T	R261Q
A/Yokohama/150/2017	3C.2A3	T135K, S144K, R150K	Null	Null	N121K	Y94H
A/Yokohama/138/2017	3C.2A4	R142G, S144R	Q197H	D53N	N171K	Null

^a^No mutation.

**Figure 4:**
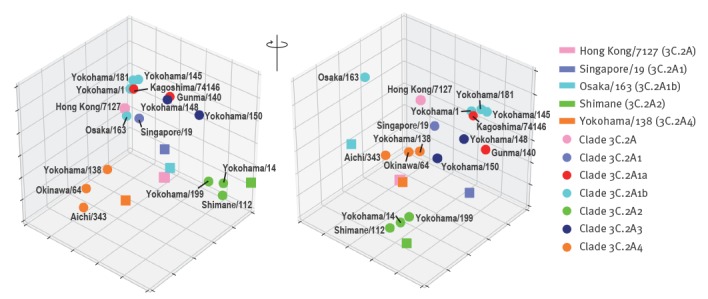
An antigenic 3D map of influenza A(H3N2) viruses, Yokohama, 2016/17 and 2017/18 influenza seasons

Using the five kinds of ferret antisera and the 16 isolates, we performed an in vitro virus neutralisation assay based on the concept of plaque reduction (Table 1). We then constructed an antigenic map to visualise these neutralisation data. This map revealed that clade 3C.2A2 (green circles) and 3C.2A4 (orange circles) viruses differed antigenically from other clades (Figure 4). Importantly, clade 3C.2A1b viruses (cyan circles) possessing 135T (A/Osaka/163/2017) differed antigenically from clade 3C.2A1b viruses possessing 135K/N (A/Yokohama/1/2018, A/Yokohama/181/2017 and A/Yokohama/145/2017) and clade 3C.2A1a and 3C2A3 viruses. The HA sequence data and antigenic analysis suggest that the amino acid substitution E62G in the HA of A/Yokohama/181/2017 and A/Yokohama/1/2018 (clade 3C.2A1b) may be responsible for the antigenic difference between A/Yokohama/181/2017 or A/Yokohama/1/2018 (clade 3C.2A1b) and Singapore/19 (clade 3C.2A1). Similarly, amino acid differences at positions 131, 142, 212 and 261 or positions 53, 94, 121, 135 and 197 are likely responsible for the antigenic difference between Shimane/112 (clade 3C.2A2) and the other tested clades or between Yokohama/138 (3C.2A4) and clades 3C.2A, 3C.2A1a, 3C.2A1b and 3C.2A3; of these amino acid changes, those at position 135 affected N-glycosylation (Tables 1 and 2; Figure 4). Moreover, loss of the N-glycosylation site at positions 133–135 due to the T135K/N mutation that was detected in some clade 3C.2A1b viruses was responsible for the antigenic difference from clade 3C.2A1b viruses possessing 135T. Although it is not yet clear which amino acid mutation(s) is required for the antigenic change, our data clearly show that antigenically distinct A(H3N2) viruses co-circulated in Yokohama, Japan during the 2016/17 and 2017/18 influenza seasons.

An antigenic 3D map was generated from the neutralisation data in Table 1. Viruses are represented as circles and antisera as squares. Strains are coloured based on the clades. The grid indicates one unit of antigenic distance, a twofold dilution in neutralisation titre.

## Discussion

Here, we showed that genetically and antigenically distinct A(H3N2) viruses co-circulated in Yokohama, Japan during the 2016/17 and 2017/18 influenza seasons. In London and Thailand, two subclades of A(H3N2) viruses, clades 3C.2A1 (denoted these as groups I to III in their study [20]) and 3C.2A3 (denoted as clade 3C.2A2 or group IV in their study [21]), were mainly responsible for the epidemics in these locations. In some cases in Thailand, A(H3N2) viruses of clade 3C.2A4 (the authors denoted these as group V) were also detected [21]. These reports, together with our results, suggest that multiple clades of A(H3N2) viruses circulated during this recent influenza epidemic. Indeed, co-circulation of multiple subclades of A(H3N2) virus, including clades 3C.2A1a, 3C.2A1b, 3C.2A2, 3C.2A3 and 3C.2A4, was also found by sequence analysis of Australian samples during the 2017 season and was documented in an early report of the 2017/18 influenza season in Canada [22,23]. In Taiwan, clade 3C.2A3 viruses were more commonly isolated from patients with severe infections; clade 3C.2A4 viruses tended to be isolated in outbreaks [24]. Although the seven residues of HA responsible for major antigenic cluster transition [5] were not changed, the antigenicity of representative isolates of clades 3C.2A2 and 3C.2A4 differed from that of isolates of other clades. Furthermore, N-linked glycosylation sites of HA have frequently changed in recent years, which has likely affected the antigenicity of the viruses [25]. In particular, the N-glycan at positions 158‒160 (NYT), which is conserved among isolates of clade 3C.2A, likely affects antigenicity because it sterically hides the residues at positions 156, 158 and 159 that are responsible for major antigenic cluster transition [5]. These reports, together with our results, show that multiple clades of A(H3N2) viruses possessing distinct antigenicity co-circulated during the 2016/17 and 2017/18 influenza seasons worldwide. In these influenza seasons, clade 3C.2A virus was used for vaccines (http://www.who.int/influenza/vaccines/virus/recommendations/en/) and clade 3C.2A1b and 3C.2A2 viruses dominated during the 2017/18 influenza season. Although the antigenicity of clade 3C.2A viruses was similar to that of clade 3C.2A1b viruses, it differed from that of clade 3C.2A2 viruses, indicating that the vaccine based on the clade 3C.2A virus did not match the circulating clade 3C.2A2 viruses in the 2017/18 influenza season. The WHO recommended the clade 3C.2A1 virus as the vaccine seed virus for the 2018 southern and 2018/19 northern hemisphere influenza season. During the 2018 southern hemisphere influenza season, the clade 3C.2A1b and 3C.2A2 viruses continued to dominate (https://nextstrain.org/flu/seasonal/h3n2/ha/3y). Whether the antigenicity of the viruses circulating in the 2018/19 northern hemisphere influenza season matches that of the clade 3C.2A1 viruses remains to be seen.
